# Preliminary evidence for a role of the adrenergic nervous system in generalized anxiety disorder

**DOI:** 10.1038/srep42676

**Published:** 2017-02-15

**Authors:** Xiaobin Zhang, Joanna Norton, Isabelle Carrière, Karen Ritchie, Isabelle Chaudieu, Joanne Ryan, Marie-Laure Ancelin

**Affiliations:** 1 Inserm, U1061, Montpellier, France; 2 Univ Montpellier, Montpellier, France; 3 Tianjin Mental Health Center, Tianjin, China; 4 Faculty of Medicine, Imperial College, London, UK; 5 Disease Epigenetics Group, Murdoch Children’s Research Institute, and Department of Paediatrics, The University of Melbourne, Parkville, Australia

## Abstract

Generalized anxiety disorder (GAD) is a common chronic condition that is understudied compared to other psychiatric disorders. An altered adrenergic function has been reported in GAD, however direct evidence for genetic susceptibility is missing. This study evaluated the associations of gene variants in adrenergic receptors (*ADRs*) with GAD, with the involvement of stressful events. Data were obtained from 844 French community-dwelling elderly aged 65 or over. Anxiety disorders were assessed using the Mini-International Neuropsychiatry Interview, according to DSM-IV criteria. Eight single-nucleotide polymorphisms (SNPs) involved with adrenergic function were genotyped; adrenergic receptors alpha(1A) (*ADRA1A*), alpha(2A) (*ADRA2A*), and beta2 (*ADRB2*) and transcription factor *TCF7L2*. Questionnaires evaluated recent stressful life events as well as early environment during childhood and adolescence. Using multivariate logistic regression analyses four SNPs were significantly associated with GAD. A 4-fold modified risk was found with *ADRA1A rs17426222* and *rs573514,* and *ADRB2 rs1042713* which remained significant after Bonferroni correction. Certain variants may moderate the effect of adverse life events on the risk of GAD. Replication in larger samples is needed due to the small case number. This is the first study showing that *ADR* variants are susceptibility factors for GAD, further highlighting the critical role of the adrenergic nervous system in this disorder.

Generalized anxiety disorder (GAD) is one of the most common chronic and debilitating anxiety disorders in older adults, with lifetime prevalence estimates ranging between 5 and 13%^ ^1,2. Previous family and twin studies suggest a substantial genetic contribution to GAD of up to 30%^ ^3,4, yet there is a paucity of studies in this area, and none in the elderly. The rare studies have examined principally neurotransmitter-related genes, and have reported relatively small effects and associations with a number of other psychiatric disorders too^5^,^6^. A targeted strategy driven by pathobiochemical mechanisms could be better suited for hypothesis-testing regarding the specific involvement of genes susceptible to play a key role in the disease etiology. Our recent findings have shown that in the elderly, GAD has specific clinical characteristics, which are distinct from other psychiatric disorders^2^,^7^. GAD in later life does not represent the continuing chronic course of early onset illness either, but has a clinical presentation distinct from GAD in younger population with age-specific predictors such as metabolic disorders and chronic diseases (*e.g.* adiposity, respiratory disorders, arrhythmia and heart failure, and cognitive impairment)^7^. Anxiety disorders are frequently triggered by stressful life events^8^ and our recent data has also shown that adverse life events including early events, were predictors of GAD in the elderly^7^. This longstanding vulnerability suggests possible biological origins related to dysfunction of the stress systems, especially the adrenergic/noradrenergic systems^7^.

Pathological abnormalities in the (nor)adrenergic system have been reported in certain stress-related psychiatric disorders, *e.g.* posttraumatic stress disorder (PTSD) and panic disorder, and preliminary evidence of pathological changes in the noradrenergic system in GAD have recently been proposed (see for review^9^). For example, adolescents with GAD have been shown to have increased plasma levels of norepinephrine after stress^10^. A direct role of adrenergic receptors (ADRs) in GAD however, has not been examined previously. ADRs belong to the G-protein-coupled receptor (GPCR) family. They are expressed in the brain and are involved in a range of processes including signal transduction of adrenaline, noradrenaline and to a lesser extent dopamine^11^. A possible role of ADRs is also supported by the anxiolytic activity of several antiadrenergic agents in GAD patients^12^,^13^, suggesting the possibility that *ADR* genetic variants could play a role in GAD.

In this study, we have investigated key *ADR* genetic variants in adrenergic-related pathways to determine their association with GAD in community-dwelling elderly, and whether lifetime stress could moderate the associations.

## Subjects and Methods

### Participants

Community-dwelling participants aged 65 years and over were recruited by random selection from electoral rolls of the Montpellier district between 1999 and 2001 as part of the ESPRIT study of neuropsychiatric disorders in French elderly^14^. Of the people initially contacted, 27.3% refused to participate and were replaced by another participant drawn randomly from the same electoral division.

### Ethics statement

All participants provided written informed consent to participate in the study, prior to the experimental protocol, and approved by the National Ethics Committee (CPP Sud Ouest et Outre Mer I France, #2007.10.23.v3 and ID RCB: 2007-A 00281-52, n°DGS2007-0477) conforming to the Declaration of Helsinki. The methods in the current study were carried out in accordance with the approved guidelines.

Persons with dementia at base-line (n = 70) were excluded from the present study but there was no other exclusion criteria. Dementia was diagnosed by a neurologist as part of a standardized examination and validated by a panel of independent neurologists according to DSM-IV revised criteria as described previously^7^,^15^. Of the 2189 non-demented participants recruited to the study, 1068 provided buccal mucosa samples for genotyping. Forty-one participants were excluded from the current analysis because they were not assessed for GAD at baseline and 183 were missing covariate data (139 of these participants having not completed the childhood questionnaire). This left 844 participants for the analysis. Compared with the participants included in the analysis, those excluded were older (t = 10.76, p < 0.001), with a lower education level (χ^2^ = 39.4, df = 1, p < 0.001), and were more likely to be overweight (χ^2^ = 18.0, df = 1, p < 0.0001), with cognitive impairment (χ^2^ = 47.1, df = 1, p < 0.0001), hypertension (χ^2^ = 11.5, df = 1, p = 0.0007), ischemic pathologies (χ^2^ = 16.3, df = 1, p < 0.0001) and phobia (χ^2^ = 6.4, df = 1, p = 0.01), and to be treated with psychotropic medication (χ^2^ = 10.5, df = 1, p = 0.001).

### Socio-demographic and clinical variables

The standardized interview included information regarding socio-demographic characteristics, physical health, medical history of the participants and current medications. Information was also collected on the history of ischemic pathologies, arrhythmia and heart failure, as well as other chronic disorders (respiratory and thyroid disorder, osteoporosis, and recent cancer). Clinical examinations were also conducted^2^,^7^. Global cognitive function was assessed with the Mini-Mental State Examination (MMSE) and cognitive impairment was defined as a score of less than 26^ ^16. Exposure to recent adverse life events during the past year was assessed using the Gospel Oak questionnaire^17^. This 12-item list of major life events included bereavement, rupture of close relationships, severe illness and serious financial or judicial problems. A retrospective self-report questionnaire examining a large range of traumatic experiences during childhood and adolescence was completed at the second follow-up assessment (after 4-years) as described previously^18^. Low affective support was defined as having reported five or less protective factors from a list of eight that included mother, father or adult friend affection, having a happy childhood, a ‘normal’ education, parents perceived as doing their best, feeling happy at school, and raised by both parents^7^.

### Psychiatric Diagnosis

The diagnosis of psychiatric disorders (GAD, social phobia, specific phobia and agoraphobia, panic disorder, obsessive compulsive disorder, PTSD, and major depression) was made by psychologists and psychiatric nurses according to DSM-IV criteria and using the Mini-International Neuropsychiatric Interview (MINI, French version 5.00) as described previously^7^. The interviewers were initially trained using video recordings of interviews by the clinicians responsible for the development of the French version of the MINI. The interviewers then administered the MINI over a 3 month period under the supervision of psychiatrists from the Department of Adult Psychiatry at Montpellier University Hospital. Dual interviews were randomly conducted to minimize interviewer drift. During this training period, false negatives were rare, in agreement with the high negative predictive value of the MINI for GAD^19^,^20^. In the Esprit study, only positive cases were thus reviewed by a panel of independent psychiatrists to validate the initial diagnosis as described previously^7^,^21^. The psychiatrists reviewed cases independently in accordance with DSM-IV criteria, using all information provided by the MINI participant responses as recorded by the interviewers, all other available clinical data, and if necessary after consultation with the participant’s general practitioner. Consensus agreement was subsequently reached for all cases. The MINI is a standardized and structured diagnostic examination validated within the general population setting^19^ which uses a nonhierarchical case-identification procedure, thus permitting the diagnosis of psychiatric comorbidities. GAD was established using the current definition implying the presence of symptoms for at least six months^22^.

### *ADR* Genotyping

We selected the most commonly studied *ADR* polymorphisms that have been associated with physiological stress response related to dysfunction of the adrenergic system and other disorders. The α1A-adrenergic receptor (*ADRA1A*) gene is located in chromosomal region 8p21.2 and the chosen SNPs include *rs3808585* in the promoter region and the three intronic SNPs *rs573514, rs4732682* and *rs17426222*. The *ADRA1A* gene has been found to be a vascular risk factor, as well as being related to obesity, attention-deficit hyperactivity disorder, and schizophrenia^23^,^24^,^25^,^26^. The α2A-adrenergic receptor (*ADRA2A*) gene is located in chromosomal region 10q25.2 and chosen SNPs include *rs1800544* in the promoter region and *rs11195419* located in the 3′ untranslated region. The *ADRA2A* gene has been associated with the risk of schizophrenia^27^, Tourette syndrome^28^, and suicidal ideation^29^, as well as high blood pressure^30^. The closely positioned *rs7903146* variant is also located in region 10q25.2 in intron 3 of the gene transcription factor 7-like 2 (*TCF7L2*). This gene encodes a high mobility group box-containing transcription factor that plays a key role in the Wnt signaling pathway influencing the transcription of a large number of genes including *GPCRs,* thereby implicated in a large variety of diseases^31^,^32^. This SNP has been linked with *ADR* variants^33^ and was associated with schizophrenia^34^, adiposity and type 2 diabetes^35^,^36^. The β2-adrenergic receptor (*ADRB2*) gene is an intronless gene with single exon in chromosomal region 5q31-32. The most extensively studied functional polymorphism is *rs1042713*, a common non-synonymous SNP resulting in an amino acid substitution (otherwise referred to as Arg16Gly). This variant has been associated with vascular response to stress, as well as alterations in cognitive function and brain white matter integrity, autism, PTSD^37^,^38^,^39^,^40^,^41^.

DNA was extracted from buccal mucosa samples collected during the second follow-up, and then stored at −80 °C. *ADR* genotyping was performed by LGC genomics, Hoddesdon, UK using their KASP SNP genotyping system as described previously^42^,^43^. KASP is a competitive allele-specific PCR incorporating a FRET quencher cassette, which has an error rate of less than 0.3%. The amplified PCR products were analyzed by fluorescence scanning in a BMG labtech Pherastar scanner and the results were interpreted with KlusterCaller 1.1 software. All SNPs except *rs180054* had also been separately genotyped by the Lille Genopole (http://www.genopole-lille.fr/spip/) using DNA extracted from blood samples collected at baseline. Both genotyping data were available for one third of the sample. The results of genotyping from buccal- and blood-cell derived DNA were identical, which helped verify the accuracy of the data. Prior genotyping data also indicated that less than 1% of the participants were non-Caucasian, and these participants were excluded from the present analyses (unpublished data and^44^).

### Statistical analysis

Chi-squared tests were used to compare the distribution of *ADR* genotypes with those expected under the Hardy-Weinberg equilibrium. Associations between *ADR* polymorphisms and current GAD were assessed using multivariate logistic regression models adjusted for age and sex. Covariates that were found to be significantly associated with GAD and which could be potential mediating factors, *e.g.* body mass index (BMI) and major depression, were also considered. Interactions with adverse life events were also examined in *post-hoc* analyses, given prior evidence to suggest possible independent group effects^39^. SAS (version 9.4, SAS institute, NC, USA) was used for all of the statistical analysis and all tests were two-tailed, with the significance level of p < 0.05. Given that eight genetic variants were investigated, the level of significance after Bonferroni-correction was set at p < 0.0063.

## Results

### Participant characteristics

Characteristics of the 844 participants (57.9% females) are summarized in Table 1. The mean age (SD) of the sample was 71.5 (4.5) years, with no significant differences between participants with and without GAD (p = 0.44). The 6 month prevalence of GAD was 4.4%. In terms of comorbidity, 13.5% of GAD cases also had major depression and 32.4% phobia.

### *ADR* polymorphisms and GAD

The *ADR* genotype frequencies were not significantly different from those predicted by Hardy-Weinberg equilibrium (p > 0.10 for all SNPs). For *rs3808585, rs1800544* and *rs11195419*, the frequency of the homozygotes for the minor allele was very low (less than 7%), and they were thus combined with the heterozygotes for analysis (Table 2). In logistic regression analyses adjusted for age and sex, two *ADRA1A* SNPs were associated with current GAD. The homozygotes for the minor alleles of *rs17426222* and *rs573514* had a more than 4-fold increased odds of GAD compared with homozygotes for the major allele (p = 0.003 and 0.005, respectively), which remained significant after Bonferroni correction. There was weaker evidence to suggest that minor allele carriers of *ADRA1A rs4732682* had a decreased risk of GAD compared to those homozygous for the major allele (CT/TT: OR = 0.47, 95% CI = 0.24–0.92, p = 0.027). No significant associations were found with the two *ADRA2A* variants. The TT homozygote of *TCF7L2 rs7903146* was associated with a 3.2-fold increased risk of GAD. In contrast to the other SNPs which showed some evidence of a dose-response, for *ADRB2 rs1042713* it was the heterozygous that were less likely to have GAD compared to the AA homozygotes. In multivariate models further adjusted for potential mediating factors, *i.e.* BMI and major depression, the same pattern of associations was found and for *ADRB2 rs1042713* the association was significant with a p-value lower than the corrected level of significance (see Supplementary Table S1).

### *ADR* polymorphisms and phobia

In this elderly population, phobia prevalence and comorbidity with GAD are relatively high in contrast with other psychiatric disorders (see Table 1). *Post-hoc* analyses were then performed to determine whether the SNPs associated with GAD were also associated with phobia (in the absence of GAD). Despite a much higher number of phobia cases than GAD, no significant associations were observed (Table 3).

### Possible role of adverse life events

In *post-hoc* analysis, given previous findings in the literature, we tested the possibility that some adverse life events modified the association between these variants and GAD. Regarding recent events, we found an interaction with *TCF7L2* (p = 0.05). The subjects with the minor T allele of *rs7903146* who reported recent adverse events had a 3.8-fold increased risk of GAD (OR = 3.78, 95% CI = 1.46–9.82, p = 0.006, n = 256) and further adjusting for past GAD episodes did not modify the association (data not shown). This was not found for those not reporting recent trauma (OR = 0.65, 95% CI = 0.15–2.80, p = 0.56, n = 212). Similarly, the T allele was also associated with a more than 5 times higher risk of GAD in the participants having reported low affective support during childhood (OR = 5.34, 95% CI = 1.01–28.3, p = 0.049, n = 68), not found among those who did not (OR = 1.50, 95% CI = 0.61–3.67, p = 0.37, n = 400). The AG heterozygotes of *ADRB2 rs1042713* were associated with lower odds of GAD only in the subjects who did not report low affective support during childhood (OR = 0.16, 95% CI = 0.05–0.56, p = 0.004, n = 322).

## Discussion

As the first genetic association study of GAD in the elderly population, we have shown that variants of the *ADR* genes are susceptibility factors for GAD, but not the other major late-life anxiety disorder, phobia. We found strong associations (4-fold modified risk) which remained after correction for multiple testing and were not mediated by other health-related factors. Our data thus support the critical role of the adrenergic nervous system in GAD and possible moderating effect of *ADR* genes in response to stressful adverse life events.

ADRs mediate action in the sympathetic nervous system through binding of catecholamines, essentially epinephrine and norepinephrine, as well as exogenous administered drugs, including alpha and beta antagonists^11^. ADRs play a pivotal role in neuroendocrine communication and are involved in the neuroendocrine control of the stress response^45^. The α1-ADRs signal mainly via G proteins of the G_q/11_ family coupling to activation of a phospholipase C and resulting in increased intracellular calcium, α2-ADRs couple to the G_i_ proteins to inhibit adenylate cyclase and subsequently the formation of cAMP, whereas β-ADRs mainly couple to G_s_ proteins to activate adenylate cyclase and cAMP production and notably, isoforms of some of the ion channels^40^. Only a limited number of small studies have addressed the role of genetic variants in the signaling cascades of ADRs^40^ and although α- and β- blockers of ADRs could be effective in treating anxiety and physiological symptoms of GAD, the relevant contribution of *ADR* variants to these effects remains largely unknown^46^.

In our study, *ADRA1A* and *ADRB2* variants were most strongly associated with GAD. No functional promoter polymorphisms have so far been identified for *ADRA1A*^40^ and the two variants associated with GAD in our study are intronic. It is possible that they could influence gene function through alternative splicing resulting from different isoforms expressed in brain^47^, however this hypothesis remains to be tested. Conversely, the *ADRB2 rs1042713* variant in the coding region appears to be functionally significant as it affects receptor down-regulation and agonist-induced ADRB2 desensitization, and as well as antagonist (beta-blockers) medication response and vascular reactivity^48^,^49^,^50^. Decreased sympathetic responsiveness, *i.e*. lower-efficiency transcription and thus lower expression of ADRB2 could protect against negative biological consequences of chronic activation of (nor)adrenergic systems in adversity exposure^39^.

While no previous studies have investigated the role of *ADR* genetic variants in GAD or phobia, associations have been reported with vascular response to stress as well as certain psychiatric disorders, *i.e.* schizophrenia and PTSD^23^,^39^,^40^. Interestingly, comorbidity and overlap between schizophrenia and anxiety have been found previously, as well as evidence for shared genes which could primarily have to do with reactivity and stress response^51^. In line with our finding that *ADRA1A* variants were associated with GAD, genetic variants in the promoter region of *ADRA1A* have been associated with schizophrenia in a Basque (Spanish) population^23^, although this failed to be replicated in a Chinese population^52^. A number of studies have also reported an association between *ADR* genes and obesity-related phenotypes including in schizophrenia patients^25^,^53^. Importantly in the present study, the associations with GAD were not mediated by BMI. We also observed an association between *TCF7L2 rs7903146* and GAD. *TCF7L2* can notably be activated by β2 adrenoreceptor agonists^33^ and can also influence the transcription of a number of other *GPCRs*, thereby implicated in a large variety of diseases, especially diabetes, through however, uncharacterized mechanisms^31^,^32^,^35^. In our study, the association with GAD remained significant after controlling for diabetes (data not shown). The direct mechanism linking this SNP with GAD is not known but it has also been associated with schizophrenia^34^. Conversely, we did not find significant associations between the two *ADRA2A* variants and GAD or phobia. A number of prior studies have examined the associations between *ADRA2A* variants and depression or schizophrenia but they also failed to find significant associations^27^,^54^,^55^,^56^.

*Rs1042713*, one of the two non-synonymous common SNPs in *ADRB2* (the other is *rs1042714* which is in strong linkage disequilibrium) is the most frequently studied variant in response to stress. Recently, Liberzon *et al*.^39^ examined the associations between *ADR* variants and PTSD in trauma-exposed adults. They did not identify any significant main effect surviving multiple correction. However, they found significant interactions between *ADRB2 rs2400707* and childhood adversity on PTSD, as well as nominally significant interactions with six other SNPs in the *ADRB2* locus, all in high linkage disequilibrium with *rs2400707*, including *rs1042713*^39^. In our study, we found a significant association between *ADRB2 rs1042713* and GAD, the risk of GAD appeared lower in the subjects who did not report low affective support during childhood. This association was specific to the heterozygotes consistent with an overdominant mode of effect^49^. A heterosis effect may occur in up to 50% of all gene associations, particularly in European people^57^ and it has already been reported with *ADRB2 rs2400707* in asthma^49^ and lung function^58^. This may also explain the conflicting findings which have been reported in association studies on vascular factors or asthma, depending on allele grouping (either G or A)^48^. An interaction between the wild type and mutant protein isoforms resulting in a heterodimeric receptor with optimal properties compared to that of the wild type or mutant homodimers has been proposed as a possible mechanism for heterozygote advantage^58^. Other explanations could be related to interactions with other genes or variants (as reported with *ADRB2* in asthma^49^), environmental factor, or age-related factors (as observed for *rs1042713* and cognitive function^37^) causing a hidden stratification of the sample such that the anxiety phenotype would be associated with either one set of homozygote subjects or the alternate homozygote set^57^.

GAD is often comorbid with other affective disorders and previous family studies suggest overlapping genetic risk factors between some disorders. We did not find significant associations between *ADR* variants and phobia despite its high prevalence in this elderly population. Our previous data indicated that whereas GAD and phobia cases shared some characteristics, they were clinically and etiologically different. GAD was more commonly associated with certain metabolic and somatic illnesses^2^, which are more specifically related to the autonomic nervous system and the (nor)adrenergic system^7^. GAD is considered to be a disorder of emotional distress, but phobia a fear-based anxiety disorder^59^. PTSD has elements of dysphoria as well as fear-based anxiety, and a recent study showed that PTSD’s dysphoria factor was uniquely related to GAD. Interestingly, significant interactions between *ADRB2* variants and either childhood adversity on PTSD^39^ or maternal stress during pregnancy on autism in children^41^ have been reported. Although speculative, this could suggest that *ADR* constitutes a vulnerability (or resilience) factor for the development of GAD following adversity, which may be shared by other stress-related disorders such as PTSD, possibly in its dysphoria dimension^59^. We also observed a significant interaction between *TCF7L2 rs7903146* and history of adversity on GAD; the participants with the minor T allele having reported recent adverse events or low affective support during childhood being at 3–5 fold higher risk of GAD compared with those who did not. So far, few specific gene-environment interactions have been described for *TCF7L2* polymorphisms, mostly in relation with metabolic dysfunction but no previous studies have examined the role of upstream factors such as stressful environment.

The main limitation of our study is the relatively small number of GAD cases, which reduced the power of our analysis. A power calculation indicates that our study had 80% power (α = 5%) to detect an OR of 4 for most of the variants, but not for the rarer alleles such as homozygotes for the minor allele of *rs4732682* where we were only powered to detect OR ≤ 0.15. This may explain the lack of significant associations with some SNPs. This is particularly the case for the analysis stratified according to stressful events and emphasizes the need for caution when interpreting these results. There are some other limitations which need to be considered. Selection bias concerned the recruitment from electoral rolls, the response rate, and the exclusion of institutionalized elderly people, which limits the generalizability of these findings. Excluding participants with missing data who were older and with poorer physical and mental health and thus more likely to be diagnosed with GAD may have decreased the overall power of the study, possibly underestimating the associations found. Bias from population stratification needs to be considered as French law prohibits collecting data related to ethnicity. However, prior genotyping data of these participants indicated that less than 1% were non-Caucasian^44^ and genotype frequencies were all within Hardy-Weinberg Equilibrium and are similar with those already published in white Europeans^24^,^27^,^34^,^38^ (and http://www.ncbi.nlm.nih.gov/projects/SNP/). Further studies are needed to replicate our findings. Despite the large size of the sample, for some SNPs the power was insufficient to examine specifically minor homozygotes. The relative sample size when stratifying GAD cases according to stressful events and genotypes also emphasizes the need for caution when interpreting these results. Furthermore, data related to life events were self-reported and retrospective, which may introduce recall bias and the temporal relationship between recent adverse events and current GAD was not assessed, but further adjusting for past GAD episodes did not change the associations.

Strengths of our study are that it was population-based and involved 844 elderly people. This is the first study to investigate and compare potential associations of several *ADR* variants with the two most prevalent anxiety disorders, GAD and phobia. Psychiatric disorders were assessed by trained staff using a standardized and validated psychiatric examination based on DSM-IV criteria. A large amount of health data were collected which allowed controlling for potential mediating factors. The genotyping system used in our study had a very low error rate, and we were able to control for accuracy through duplicate samples.

To our knowledge, this is the first study which provides evidence that *ADRA1A* and *ADRB2* variants are strongly associated with GAD in the elderly, but not shared with the other major anxiety disorder, phobia. Our data also support the possible moderating effect of *ADR* genes in response to recent or early stressful life events. Exposure to adverse events has been associated with nervous system dysfunction and marked long-term changes in brain circuitry that regulates stress reactivity. Additional investigations are needed to confirm these findings in larger population samples and investigate the functionality of the associated polymorphisms. Our findings have implications not only for research but also potentially for nosology and practice. They provide an important lead for examining the pathogenesis of GAD involving the adrenergic stress system. Findings also have potential clinical implications for GAD etiology and more largely stress-related disorders, as well as for developing novel specific preventative and intervention strategies.

## Additional Information

**How to cite this article**: Zhang, X. *et al*. Preliminary evidence for a role of the adrenergic nervous system in generalized anxiety disorder. *Sci. Rep.* **7**, 42676; doi: 10.1038/srep42676 (2017).

**Publisher's note:** Springer Nature remains neutral with regard to jurisdictional claims in published maps and institutional affiliations.

## Supplementary Material

Supplementary Table 1

## Figures and Tables

**Table 1:** Characteristics of participants according to current GAD^a^ in an elderly cohort (n = 844^b^).

Characteristic	No GAD		GAD (n = 37)		GAD *versus* no GAD		
	Mean	SD	Mean	SD			P
Age	71.55	4.47	70.97	4.10			0.44
	N	%	N	%	OR^c^	95% CI	p
Sex (female)	461	57.13	28	75.68	2.35	1.10–5.05	0.03
Education level (>5 years)	442	54.77	15	40.54	0.63	0.32–1.24	0.18
BMI (≥25 kg/m^2^)	346	42.87	8	21.62	0.42	0.19–0.93	0.03
At least one recent adverse events during the past year	463	57.37	29	78.38	2.68	1.21–5.96	0.02
Low affective support	111	13.75	13	35.14	3.28	1.62–6.67	0.001
Hypercholesterolemia (cholesterol ≥6.2 mmol/L or treated)	451	56.09	21	56.76	0.87	0.44–1.71	0.68
Hypertension (resting blood pressure ≥160/95 mm Hg or treated)	321	39.78	14	37.84	1.03	0.52–2.05	0.93
Ischemic pathologies^d^	82	10.16	4	10.81	1.35	0.46–3.99	0.59
Arrhythmia and heart failure	97	12.03	6	16.22	1.52	0.61–3.77	0.37
At least one chronic disorder^e^	469	58.12	20	54.05	0.91	0.47–1.77	0.78
MMSE (<26)	76	9.43	5	13.51	1.38	0.52–3.67	0.52
Use of psychotropic medication	80	9.91	10	27.03	3.02	1.38–6.62	0.006
Major depression	11	1.38	5	13.51	10.04	3.24–31.1	<0.0001
Anxiety disorder (without GAD)	69	8.55	12	32.43	4.40	2.11–9.37	<0.0001
Phobia	64	7.93	12	32.43	4.81	2.27–10.2	<0.0001
Post-traumatic stress disorder	1	0.12	1	2.70	n.a	n.a	n.a
Panic disorder	1	0.12	0	0.00	n.a	n.a	n.a
Obsessive compulsive disorder	4	0.50	1	2.70	n.a	n.a	n.a

^a^The diagnosis of GAD and other psychiatric disorders was performed using the MINI and according to DSM-IV criteria (see Methods).

^b^Except for hypercholesterolemia (n = 841); arrhythmia and heart failure and MMSE (n = 843); major depression (n = 837).

^c^Adjusted for age (continuous) and sex.

^d^Ischemic pathologies correspond to angina pectoris, myocardial infarction, stroke, cardiovascular surgery, and arteritis.

^e^Chronic disorders correspond to hypercholesterolemia, hypertension, diabetes, asthma, osteoporosis, thyroid disorder, and recent cancer.

n.a.: not applicable due to the low number of participants.

**Table 2:** Logistic regression analysis for the association between *ADR* polymorphisms and current GAD in an elderly cohort^a^.

*Gene*	*SNPs and genotype*		No GAD		GAD		Odds of pure phobia *versus* no GAD		
			N	%	N	%	OR^a^	95% CI	p
***ADRA1A***	*rs4732682*	CC	225	29.7	17	47.2	—	—	—
		CT	386	51.0	15	41.7	0.51	0.25–1.04	0.06
		TT	146	19.3	4	11.1	0.36	0.12–1.09	0.07
	*rs17426222*	CC	433	54.0	13	35.1	—	—	—
		CT	315	39.3	17	46.0	1.81	0.86–3.79	0.12
		TT	54	6.7	7	18.9	4.34	1.65–11.4	0.003
	*rs3808585*	CC	418	52.6	21	56.8	—	—	—
		CT/TT	376	47.4	16	43.2	0.84	0.43–1.64	0.61
	*rs573514*	TT	224	28.6	5	14.3	—	—	—
		CT	414	52.9	15	42.9	1.58	0.56–4.41	0.39
		CC	145	18.5	15	42.9	4.48	1.59–12.7	0.005
***ADRA2A***	*rs1800544*	CC	422	54.4	20	54.1	—	—	—
		CT/TT	353	45.6	17	45.9	0.97	0.50–1.89	0.94
	*rs11195419*	CC	650	82.7	29	78.4	—	—	—
		AA/AC	136	17.3	8	21.6	1.34	0.60–3.01	0.47
***TCF7L2***	*rs7903146*	CC	340	43.5	10	27.8	—	—	—
		CT	358	45.8	18	50.0	1.68	0.76–3.70	0.20
		TT	84	10.7	8	22.2	3.23	1.23–8.52	0.018
***ADRB2***	*rs1042713*	AA	110	13.8	9	24.3	—	—	—
		AG	372	46.7	9	24.3	0.28	0.11–0.73	0.009
		GG	314	39.5	19	51.4	0.73	0.32–1.67	0.45

^a^Adjusted for age and sex.

**Table 3:** Logistic regression analysis for the association between *ADR* polymorphisms and current phobia^a^.

*Gene*	*SNPs and genotype*		No phobia		Pure phobia		Odds of pure phobia *versus* no phobia		
			N	%	N	%	OR	95% CI	p
***ADRA1A***	*rs4732682*	CC	214	30.75	11	18.03	—	—	—
		CT	350	50.29	36	59.02	1.95	0.97–3.94	0.06
		TT	132	18.97	14	22.95	2.04	0.89–4.68	0.09
	*rs17426222*	CC	399	54.21	34	51.52	—	—	—
		CT	290	39.40	25	37.88	1.03	0.59–1.77	0.93
		TT	47	6.39	7	10.61	1.81	0.75–4.37	0.19
	*rs3808585*	CC	388	53.22	30	46.15	—	—	—
		CT/TT	341	46.78	35	53.85	1.30	0.77–2.18	0.32
	*rs573514*	TT	206	28.57	18	29.03	—	—	—
		CT	381	52.84	33	53.23	0.96	0.52–1.77	0.90
		CC	134	18.59	11	17.74	0.92	0.42–2.03	0.83
***ADRA2A***	*rs1800544*	CC	389	54.33	33	55.00	—	—	—
		CT/TT	327	45.67	27	45.00	0.92	0.54–1.57	0.76
	*rs11195419*	CC	597	82.46	53	85.48	—	—	—
		AA/AC	127	17.54	9	14.52	0.82	0.39–1.71	0.59
***TCF7L2***	*rs7903146*	CC	310	43.00	30	49.18	—	—	—
		CT	333	46.19	25	40.98	0.77	0.44–1.36	0.37
		TT	78	10.82	6	9.84	0.81	0.32–2.05	0.66
***ADRB2***	*rs1042713*	AA	104	14.23	6	6.23	—	—	—
		AG	339	46.37	33	50.77	1.68	0.68–4.16	0.26
		GG	288	39.40	26	40.00	1.61	0.64–4.06	0.31

^a^Adjusted for age and sex. The participants with prevalent GAD were not included in the analysis.
