# Supplementary material for: Preliminary evidence for a role of the adrenergic nervous system in generalized anxiety disorder
Source: Sci Rep. 2017 Feb 15;7:42676. doi: 10.1038/srep42676 (PMC5309880; doi:10.1038/srep42676)
Supplement: Supplementary Table 1 [file srep42676-s1.pdf]

SUPPLEMENTARY INFORMATION

Preliminary evidence for a role of the adrenergic nervous system in generalized anxiety disorder

Xiaobin Zhang, Joanna Norton, Isabelle Carrière, Karen Ritchie, Isabelle Chaudieu, Joanne Ryan & Marie-Laure Ancelin*

Supplementary Table S1. Multivariate logistic regression analysis[a] for the association between *ADR* polymorphisms and GAD

Gene	***SNPs and genotype***		**OR**	**95%CI**	**p**
ADRA1A	*rs4732682*	CC	-		-
		CT	0.50	0.24-1.04	0.06
		TT	0.38	0.12-1.19	0.10
	rs17426222	CC	-		-
		CT	1.66	0.78-3.53	0.18
		TT	3.80	1.40-10.3	0.009
	rs3808585	CC	-		-
		CT/TT	0.87	0.44-1.73	0.70
	rs573514	TT	-		-
		CC	1.43	0.50-4.08	0.50
		CT	4.42	1.53-12.8	0.006
ADRA2A	*rs1800544*	CC	-		-
		CT/TT	0.98	0.50-1.93	0.96
	rs11195419	CC	-		-
		AA/AC	1.25	0.54-2.88	0.60
TCF7L2	*rs7903146*	CC	-		-
		CT	1.65	0.74-3.69	0.22
		TT	3.13	1.16-8.46	0.024
ADRB2	*rs1042713*	AA	-		-
		AG	0.23	0.08-0.62	0.004
		GG	0.67	0.29-1.57	0.36

[a] Adjusted for age, sex, BMI and major depression
